# Supplementary material for: Epithelial-to-Mesenchymal Transition Drives Invasiveness of Breast Cancer Brain Metastases
Source: Cancers (Basel). 2022 Jun 25;14(13):3115. doi: 10.3390/cancers14133115 (PMC9264851; doi:10.3390/cancers14133115)
Supplement: Supplementary file 1 [file cancers-14-03115-s001.zip › cancers-1764791-Supplementary.pdf]

cancers

MDPI

Supplementary Figures

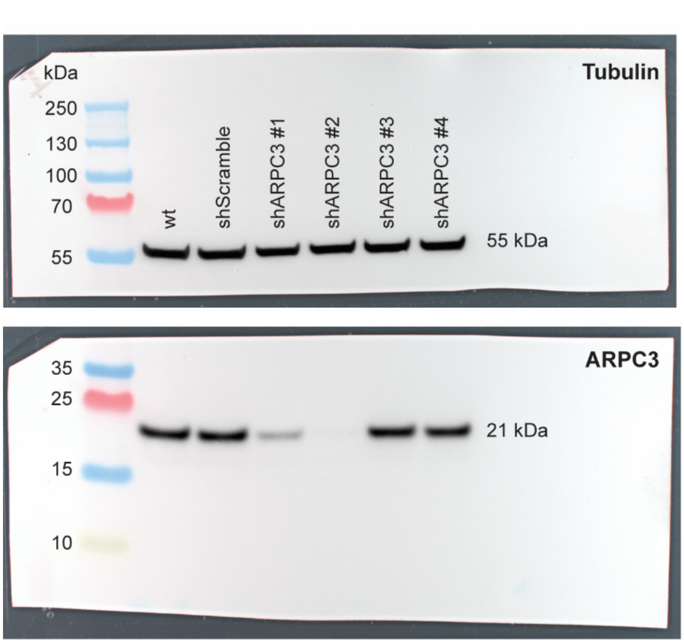

Figure S1. Western blot analysis of ARPC3 knockdown in BCBM organoids. (**A**) Uncropped Western blot showing ARPC3 expression in BCBM organoids engineered with all ARPC3 shRNA's. Tubulin was used as a loading control.

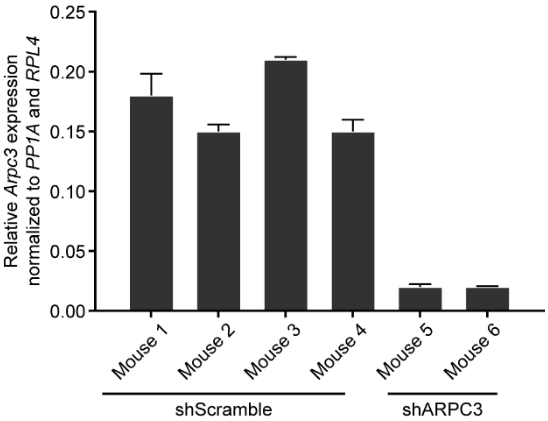

Figure S2. ARPC3 knockdown validation by RT-qPCR in recurrent tumors. Relative Arpc3 expression in recurrent tumors in mice injected with BCBM organoids enginnered with either the shARPC3 or shScramble constructs. Data are presented as mean +/- SD. n=1 mouse with 3 technical replicates.

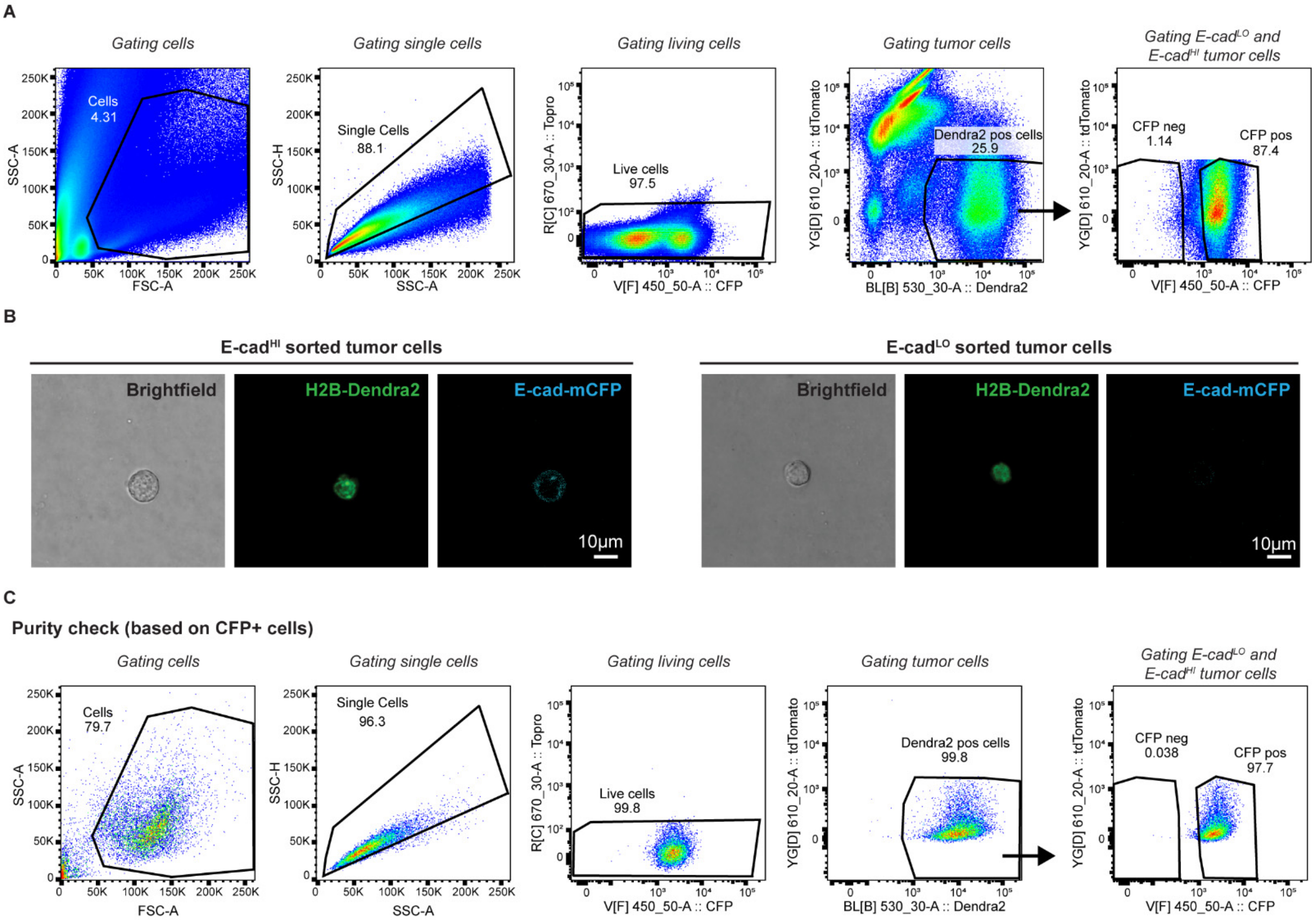

Figure S3. Gating strategy of BCBM tumors FACS (**A**) Gating strategy applied to BCBM tumors to sort EcadHI and EcadLO tumor cells. (**B**) Representative images of EcadHI and EcadLO sorted tumor cells. (**C**) Gating strategy to analyze the purity of our FACS strategy. Due to the limited amount of EcadLO cells that we could get for this experiment, we performed the purity control using the EcadHI tumor cells.

Table S1. Primer sequences.

Primer	Sequence
RPL38_F	5´-AGGATGCCAAGTCTGTCAAGA-3´
RPL38_R	5´-TCCTTGTCTGTGATAACCAGGG-3´
PPIA_F	5´-GTTCATGCCTTCTTTCACCTTCCC-3´
PPIA_R	5´-CAAATGCTGGACCAAACACAAACG-3´
Vim-F	5´-TGGTACAAGTCCAAGTTTGC-3´
Vim-R	5´-GTGAGTGACTGCACCTGTCT-3´
Snai1-F	5´-TTGTAACAAGGAGTACCTCAGC-3´
Snai1-R	5´-GAGAATGGCTTCTCACCAGTG-3´
Zeb1-F	5´-GCTGGCAAGACAACGTGAAAG-3´
Zeb1-R	5´-GCCTCAGGATAAATGACGGC-3´
Cdh1-F	5´-CAGGTCTCCTCATGGCTTTGC-3´
Cdh1-R	5´-CTTCCGAAAAGAAGGCTGTCC-3´
Cdh2-F	5´-AGCGCAGTCTTACCGAAGG-3´
Cdh2-R	5´-TCGCTGCTTTCATACTGAACTTT-3´
Fn1-F	5´-AGAGCAAGCCTGAGCCTGA-3´
Fn1-R	5´-GTCCCAGATCATGGAATCTT-3´
Zeb2-F	5´-ATTGCACATCAGACTTTGAGGAA-3´
Zeb2-R	5´-GAGTTCCAGGTGGCAGGTCAT-3´
Arpc3-F	5´-GGACACCGTCAAGATGCCG-3´
Arpc3-R	5´-GCCTTGAAGTAGTAGATGGCTT-3´
